# Morphology of the snake spectacle reflects its evolutionary adaptation and development

**DOI:** 10.1186/s12917-017-1193-2

**Published:** 2017-08-18

**Authors:** Mari-Ann Otkjaer Da Silva, Steffen Heegaard, Tobias Wang, Jacob Thorup Gade, Christian Damsgaard, Mads Frost Bertelsen

**Affiliations:** 1 Centre for Zoo and Wild Animal Health, Copenhagen Zoo, Roskildevej 38, DK-1870 Frederiksberg, Denmark; 2 Eye Pathology Section, Department of Pathology, Rigshospitalet, University of Copenhagen, Frederik V’s Vej 11, DK-2100 Copenhagen, Denmark; 3 Department of Ophthalmology, Rigshospitalet, University of Copenhagen, Nordre Ringvej 57, DK-2600 Glostrup, Denmark; 4 Zoophysiology, Department of Biosciences, Aarhus University, DK-8000 Aarhus C, Denmark

**Keywords:** Eye, Snake, Spectacle, Thickness

## Abstract

**Background:**

Covering the eye of all snakes is a transparent integumental structure known as the spectacle. In order to determine variations in spectacle thickness among species, the spectacles of 217 alcohol-preserved museum specimens of 44 species belonging to 14 different families underwent optical coherence tomography (OCT) to measure spectacular thickness. Multivariable analyses were made to determine whether family, activity period (diurnal/nocturnal) and habitat (arboreal/terrestrial/fossorial/aquatic) influenced spectacle thickness.

**Results:**

The thinnest spectacles in absolute terms were found in the Usambara bush viper (Viperidae) with a thickness of 74 ± 9 μm and the absolute thickest spectacle was found in the red-tailed pipe snake (Cylindrophiidae) which had a spectacle thickness of 244 ± 57 μm. Fossorial and aquatic snakes had significantly thicker spectacles than arboreal and terrestrial snakes. When spectacle thickness was correlated to eye size (horizontal spectacle diameter), Gray’s earth snake (Uropeltidae) had the lowest ratio (1:7) and the cottonmouth (Viperidae) had the highest ratio (1:65). Multivariable and phylogenetic analyses showed that spectacular thickness could be predicted by taxonomic family and habitat, but not activity period.

**Conclusion:**

This phylogenetically broad systematic study of the thickness of the snake spectacle showed that spectacular thickness varies greatly across snake species and may reflect evolutionary adaptation and development.

## Background

The eye of all snakes is covered by a transparent spectacle that stems from fusion of the eye lids during embryonic development [1, 2]. It is generally believed that the spectacle arose as an evolutionary adaptation to protect the eyes of fossorial early snakes [3, 4]. However, given that extant snakes thrive in a variety of habitats with some species being almost exclusively fossorial, aquatic or terrestrial, and display diverse activity patterns where some species are nocturnal and others primarily active during the day, it is reasonable to expect substantial interspecies and adaptive differences in the requirement of the spectacle in terms of providing physical protection of the eye or partaking in visual optics. In principle, the morphological adaptations of the spectacle could have developed by homology (i.e.*,* the morphology of the spectacle is predicted by the family of the snake), by convergent evolution (predicted by environment or habitat) or randomly (no pattern detected). A recent study on the eye size of colubrid snakes [5] found that eye size was not predicted by taxonomy, but was an adaptation to environment suggesting convergent evolution.

We have recently demonstrated excellent correlation between measurements of spectacle thickness collected via optical coherence tomography (OCT), a non-contact medical imaging technology where reflected light is used to produce detailed cross-sectional images of biological tissue [6] and those measured by light microscopy of formalin-fixed specimens [7].

The aim of this study was to use OCT to test the hypotheses that spectacle thickness in snakes varies among species in absolute terms and relative to horizontal spectacle diameter, and we investigated whether spectacular thickness reflects adaptation to different habitats and/or daily activity patterns or could be predicted by taxonomy.

## Methods

Snakes for this study were obtained from the Natural History Museum of Denmark (Copenhagen). All 217 specimens were fixed with an injection of formalin and subsequently stored in ethanol. Specimens had been stored for 5-100 years. Date of collection was recorded by the museum, but this information was not included as a variable in this study. The museum had identified the various species and assigned the species to a taxonomic family using the Reptile Database [8]. The habitat of each species was defined as arboreal, terrestrial, fossorial, or aquatic and assigned as nocturnal or diurnal based on the period of the day during which they were believed to demonstrate major activity. These characteristics were defined by available field guides and textbooks along with scientific literature [9–32]. Only species that primarily occupy one type of habitat were chosen to reduce the amount of habitat groups as well as avoid speculation of whether one type of habitat has a larger influence than another. The same principle was used for the activity period. Horizontal spectacular diameter was recorded as an indicator of globe size as seen in a previous study [5] by using handheld electronic callipers.

Both eyes of all snakes underwent OCT scanning (OCT Spectralis, Heidelberg Engineering) using a fitted anterior segment lens which allowed non-contact anterior eye segment recordings through laser scanning and provided images in a few seconds. Absolute thickness of the spectacle was measured on the scanning images using the built-in electronic callipers supplied by the equipment software, by taking the average of three measurements made perpendicular to the spectacle surface in the centre of the spectacle proper. A ratio between spectacle thickness and spectacle diameter was calculated in percent (spectacle thickness in μm divided by spectacle diameter in mm). This ratio will be referred to as the relative thickness of the spectacle. Measurements from the right and left eye were averaged for each individual to eliminate variability between the two sides.

Data were analysed using Microsoft Excel 2010, JMP 9.0.2 (SAS Institute Inc.), and Mesquite 3.2. Spectacular thickness was compared among snake species in absolute terms and relative to spectacle diameter using ANOVA. Multivariable analyses through linear regression were made to determine whether habitat, period of activity, or family predicted spectacle thickness. Post-hoc testing (Tukey’s test) defined the details. Ancestral states for continuous (spectacle thickness and diameter) and categorical variables (habitat types and activity period) were reconstructed using linear parsimony and maximum parsimony, respectively, using the latest phylogeny of snakes and using branch length proportional to time [33]. Phylogenetically independent contrasts analysis was used to correlate evolutionary increases in spectacle diameter and thickness [34]. Probability values (*p*) equal to or less than 0.05 were accepted as significant. All data are presented as mean ± standard deviation (SD).

## Results

**Fig. 1:**
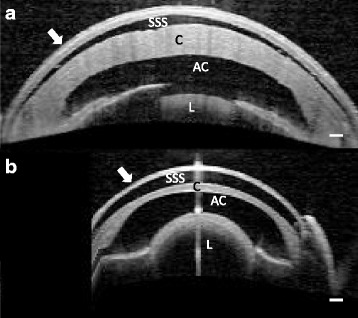
Optical coherence tomography images of the spectacle (arrow) and anterior eye segment of two snakes. Subspectacular space (SSS), cornea (C), anterior chamber (AC), lens (L). Bar = 200 μm. **a**: Green anaconda (*Eunectes murinus)*, an aquatic snake with an average spectacle thickness of 156 μm. **b**: Brown tree snake (*Boiga irregularis*), an arboreal snake with an average spectacle thickness of 102 μm

A total of 217 snakes representing 44 species were assessed (Table 1). The number of individuals examined of each species varied from 3 to 7, depending on species tested. These were defined as living in an arboreal (*n* = 39), terrestrial (117), fossorial (30), or aquatic (31) habitat; 30 were defined as nocturnal and 14 as diurnal. OCT provided clear images of the snake anterior segment (Fig. 1) that permitted measurement of spectacular thickness in all animals assessed. Mean ± SD spectacular thickness and horizontal spectacular diameter for all species examined are listed in Table 1. In absolute terms, the thinnest spectacles were measured in two nocturnal species: Usambara bush vipers (*Atheris ceratophora*) (Viperidae; 74 ± 9 μm) and the olive house snakes (*Boaedon olivaceus*) (Lamprophiidae; 74 ± 20 μm). Whereas, the thickest spectacle was measured in the fossorial, nocturnal red-tailed pipe snake (*Cylindrophis ruffus*) (Cylindrophiidae; 244 ± 57 μm). The fossorial, nocturnal Gray’s earth snake (*Uropeltis melanogaster*) (Uropeltidae) had the smallest horizontal spectacular diameter (0.7 ± 0.1 mm) and the terrestrial, diurnal amethystine python (*Morelia amethistina*) (Pythonidae) had the largest spectacular diameter (7.7 ± 0.3 mm). When spectacular thickness was correlated to horizontal spectacle diameter, Gray’s earth snake (*U. melanogaster*) (Uropeltidae) had the lowest ratio (1:7) and the aquatic, diurnal cottonmouth (*Agkistrodon piscivorus*) (Viperidae) had the highest ratio (1:65).

**Table 1:** List of species examined by optical coherence tomography with habitat, activity pattern and measurements of horizontal spectacle diameter and spectacle thickness

Family	Scientific Name	Common Name	Habitat	Activity Period	n	Spectacle Diameter (mm)	Spectacle Thickness (μm)
Acrochordidae	*Acrochordus javanicus*	Javan file snake	AQ	N	5	3.4 ± 0.4 CI95 [2.9;3.8]	155 ± 35 CI95 [112;199]
Cylindrophiidae	*Cylindrophis ruffus*	Red-tailed pipe snake	F	N	5	2.4 ± 0.5 CI95 [1.7;3.0]	244 ± 57 CI95 [173;315]
Uropeltidae	*Uropeltis melanogaster*	Gray’s earth snake	F	N	4	0.7 ± 0.1 CI95 [0.5;0.8]	100 ± 13 CI95 [78;121]
Pythonidae	*Morelia amethistina*	Amethystine python	T	D	4	7.7 ± 0.3 CI95 [7.3;8.1]	198 ± 40 CI95 [135;262]
	*Broghammerus reticulatus*	Reticulated python	T	N	5	5.8 ± 0.5 CI95 [5.2;6.4]	154 ± 28 CI95 [117;191]
Xenopeltidae	*Xenopeltis unicolor*	Sunbeam snake	F	N	5	2.4 ± 0.3 CI95 [2.0;2.8]	167 ± 36 CI95 [122;211]
Boidae	*Corallus hortulanus*	Garden tree boa	A	N	4	5.0 ± 0.7 CI95 [3.9;6.1]	150 ± 33 CI95 [98;203]
	*Chilabothrus angulifer*	Cuban boa	A	N	3	6.4 ± 0.7 CI95 [4.6;8.3]	226 ± 34 CI95 [141;311]
	*Boa constrictor*	Boa constrictor	T	N	8	4.1 ± 0.6 CI95 [3.6;4.6]	146 ± 45 CI95 [108;184]
	*Acrantophis madagascariensis*	Madagascan ground boa	T	N	3	6.4 ± 0.3 CI95 [5.6;7.2]	179 ± 51 CI95 [51;306]
	*Eryx jaculus*	Javelin sand boa	F	D	4	2.2 ± 0.1 CI95 [1.9;2.4]	119 ± 20 CI95 [88;150]
	*Eunectes murinus*	Green anaconda	AQ	D	3	6.0 ± 0.5 CI95 [4.7;7.4]	156 ± 34 CI95 [72;239]
Colubridae	*Dispholidus typus*	Boomslang	A	D	5	6.4 ± 0.5 CI95 [5.8;7.0]	185 ± 28 CI95 [151;220]
	*Boiga irregularis*	Brown tree snake	A	N	5	5.5 ± 0.2 CI95 [5.2;5.8]	102 ± 9 CI95 [87;116]
	*Ahaetulla nasuta*	Green vine snake	A	N	7	3.7 ± 0.1 CI95 [3.6;3.8]	92 ± 10 CI95 [82;101]
	*Pantherophis guttata*	Eastern corn snake	T	D	4	4.6 ± 0.2 CI95 [4.0;5.2]	93 ± 25 CI95 [54;131]
	*Lampropeltis getula*	Common kingsnake	T	N	5	4.2 ± 0.6 CI95 [3.4;5.0]	137 ± 32 CI95 [97;177]
	*Lampropeltis triangulum*	Scarlet kingsnake	T	N	4	2.9 ± 0.3 CI95 [2.4;3.3]	115 ± 29 CI95 [69;161]
	*Crotaphopeltis hotamboeia*	Red-lipped herald	T	N	5	3.3 ± 0.4 CI95 [2.7;3.8]	105 ± 17 CI95 [85;126]
	*Pituophis melanoleucus*	Eastern pine snake	F	N	3	5.4 ± 0.1 CI95 [5.2;5.5]	109 ± 17 CI95 [68;150]
Lamprophiidae	*Rhamphiophis rostratus*	Rufous beaked snake	T	D	5	4.9 ± 0.4 CI95 [4.3;5.3]	124 ± 28 CI95 [95;153]
	*Lamprophis fuliginosus*	African house snake	T	N	6	3.6 ± 0.4 CI95 [3.2;4.0]	139 ± 21 CI95 [107;172]
	*Lamprophis aurora*	Aurora house snake	T	N	4	3.2 ± 0.3 CI95 [2.6;3.7]	83 ± 32 CI95 [32;135]
	*Boaedon olivaceus*	Olive house snake	T	N	3	2.5 ± 0.2 CI95 [2.0;3.0]	74 ± 20 CI95 [24;123]
	*Lycophidion capense*	Cape wolf snake	T	N	5	1.7 ± 0.2 CI95 [1.4;2.0]	93 ± 26 CI95 [52;133]
	*Gonionotophis poensis*	Western forest file snake	T	N	5	2.6 ± 0.4 CI95 [1.7;3.5]	88 ± 21 CI95 [55;122]
	*Lycodonomorphus bicolor*	Tanganyika water snake	AQ	N	4	2.2 ± 0.3 CI95 [1.7;2.7]	125 ± 16 CI95 [99;150]
Elapidae	*Dendroaspis angusticeps*	Green mamba	A	D	4	5.1 ± 0.5 CI95 [3.7;6.5]	118 ± 15 CI95 [81;155]
	*Naja naja*	Indian cobra	T	D	4	5.5 ± 0.4 CI95 [4.6;6.6]	111 ± 10 CI95 [95;127]
	*Acanthophis antarcticus*	Common death adder	T	N	6	2.7 ± 0.3 CI95 [2.4;3.0]	97 ± 11 CI95 [85;108]
	*Aspidomorphus muelleri*	Müllers crown snake	F	N	4	2.5 ± 0.7 CI95 [0.7;4.2]	99 ± 24 CI95 [60;138]
	*Hydrophis ornatus*	Ornate reef seasnake	AQ	D	4	3.2 ± 0.2 CI95 [2.9;3.5]	188 ± 29 CI95 [141;234]
	*Hydrophis platurus*	Yellowbelly seasnake	AQ	N	6	3.4 ± 0.2 CI95 [3.2;3.7]	128 ± 33 CI95 [92;163]
Homalopsidae	*Fordonia leucobalia*	Crab-eating watersnake	AQ	N	5	1.6 ± 0.2 CI95 [1.3;1.8]	144 ± 27 CI95 [111;177]
Viperidae	*Trimeresurus albolabris*	White-lipped tree viper	A	D	5	3.4 ± 0.3 CI95 [3.0;3.7]	81 ± 26 CI95 [49;113]
	*Atheris ceratophora*	Usambara bush viper	A	N	6	3.7 ± 0.5 CI95 [3.1;4.2]	74 ± 9 CI95 [64;84]
	*Vipera berus*	Adder	T	D	9	3.1 ± 0.6 CI95 [2.5;3.8]	84 ± 24 CI95 [55;113]
	*Bitis arietans*	Puff adder	T	N	7	4.3 ± 0.5 CI95 [3.7;4.8]	104 ± 17 CI95 [89;120]
	*Agkistrodon piscivorus*	Cottonmouth	AQ	D	4	5.2 ± 0.6 CI95 [4.1;6.1]	80 ± 14 CI95 [58;102]
Aniliidae	*Anilius scytale*	Coral cylinder snake	F	N	5	1.2 ± 0.3 CI95 [0.8;1.5]	133 ± 26 CI95 [91;175]
Natricidae	*Thamnophis sirtalis*	Garter snake	T	D	7	3.8 ± 0.7 CI95 [3.2;4.4]	86 ± 17 CI95 [70;102]
	*Natriciteres olivacea*	Olive marsh snake	T	D	6	2.3 ± 0.4 CI95 [1.8;2.8]	86 ± 29 CI95 [55;117]
	*Rhabdophis subminiatus*	Red-necked keelback	T	N	7	3.6 ± 0.2 CI95 [3.5;3.8]	91 ± 15 CI95 [77;105]
Pseudo-xenodontidae	*Plagiopholis nuchalis*	Assam mountain snake	T	N	3	2.4 ± 0.5 CI95 [1.1;3.6]	88 ± 17 CI95 [46;131]

Habitat was defined as *A* arboreal, *T* terrestrial, *F* fossorial, or *AQ* aquatic. Active period was defined as *D* diurnal, *N* nocturnal. *n* number of animals examined. Parameters shown are mean ± standard deviation and 95% confidence intervals (CI95)

For spectacular thickness, multivariable analysis identified taxonomic family as a significant parameter (*p* < 0.0001). Burrowing snakes of the Cylindrophiidae had the thickest spectacles and Viperidae the thinnest spectacles. Habitat was also significantly correlated with absolute spectacular thickness (*p* = 0.0083), but not spectacular thickness relative to horizontal spectacular diameter (*p* = 0.5795). Daily period of activity was not significantly correlated with absolute or relative spectacular thickness (*p* = 0.7024 and 0.1653, respectively). Terrestrial snakes differed significantly from fossorial and aquatic snakes in both absolute and relative spectacle thickness. They possessed the thinnest spectacles and the highest ratio. In contrast, there were no significant differences between arboreal and terrestrial snakes. Arboreal snakes showed no significant differences to any of the other groups in absolute terms, but differed significantly from fossorial and aquatic species in relative terms by having a ratio similar to the terrestrial snakes. There was no significant difference between fossorial and aquatic snakes on an absolute spectacle thickness level, but relative to spectacle diameter, fossorials had a significantly smaller ratio.

Multivariable analysis showed that all three parameters: family (*p* < 0.0001), habitat (*p* < 0.0001) and activity (*p* < 0.0001) were correlated with eye size. The family with the significantly largest spectacle diameters was the Pythonidae, whereas the Uropeltidae displayed the smallest spectacle diameters. Arboreal snakes had the significantly largest eyes and the fossorial snakes had the smallest eyes. There was no significant difference between the size of the eyes of terrestrial snakes and aquatic snakes. Diurnal snakes had significantly larger horizontal spectacle diameter than nocturnal snakes.

**Fig. 2:**
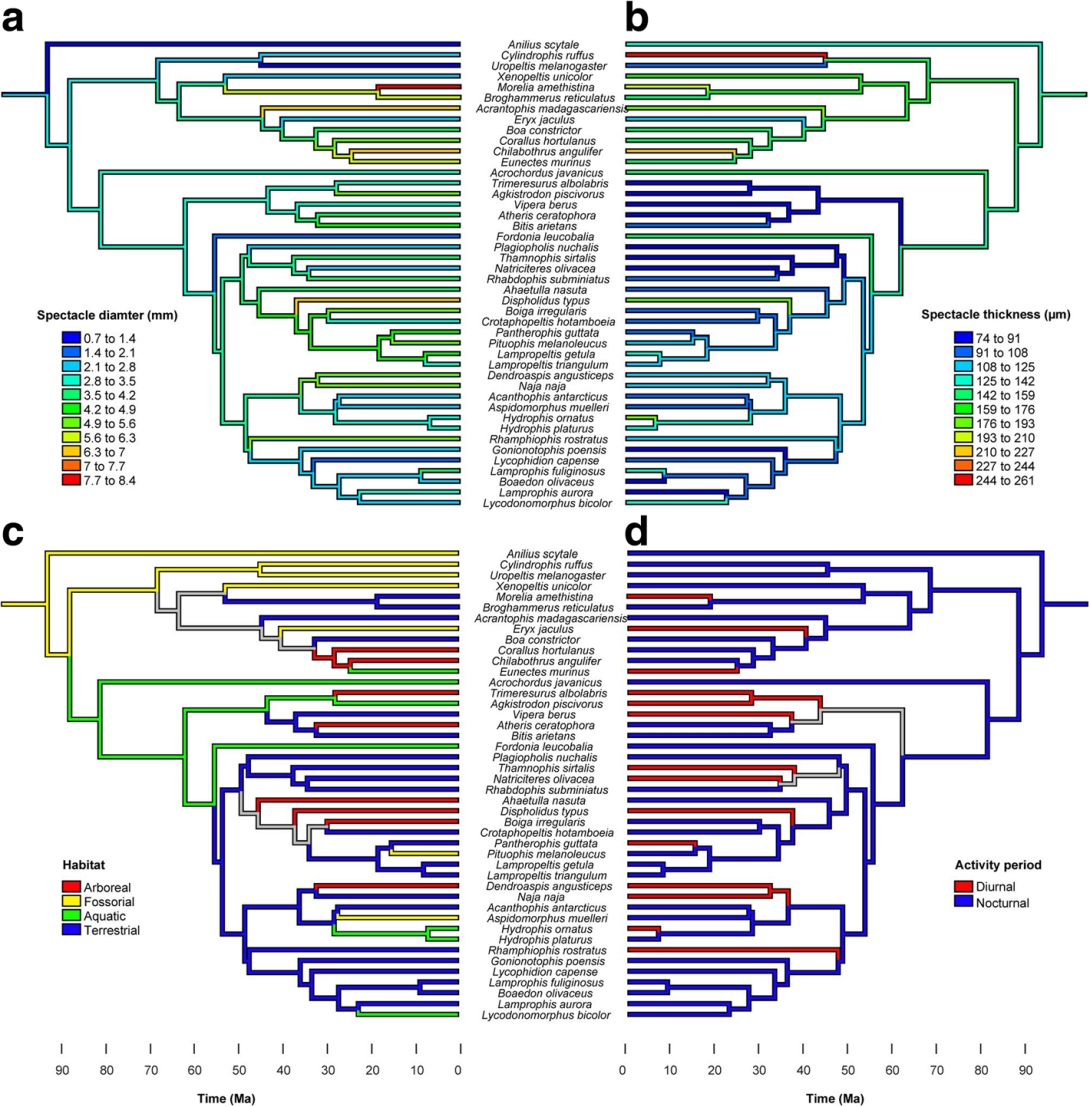
Evolution of spectacle morphology, habitat type, and activity period in snakes. Plots show measured (tips) and reconstructed (internal branches) values of spectacle diameter (**a**), spectacle thickness (**b**), habitat type (**c**), and activity period (**d**) plotted on the phylogeny of snakes (Ma: million years). Internal branch values were reconstructed using linear parsimony in **a** and **b**, and maximum parsimony in **c** and **d**. Grey branches in c and d represent branches were it was not possible to resolve habitat and activity period

Overall, the phylogenetic analyses were consistent with the conclusions from the multivariate analysis. They showed that ancestral snakes were most likely fossorial and nocturnal with small eyes (1.2–3.4 mm) and moderately thick spectacles (133–155 μm) (Fig. 2a, b). Diurnal activity period originated 10 times independently (Fig. 2d ), and was only associated with evolutionary increases in absolute spectacular thickness in one of these events (Fig. 2b), but was associated with increases in spectacle diameter in only some of these cases (Fig. 2a). Habitat changed 18 times within the 44 species examined, why ancestral state reconstruction of habitat type was not robust on all branches within the phylogeny (Fig. 2c). Still, the phylogenetic reconstructions did confirm three of the central conclusions from the multivariate analysis by showing 1) increases in spectacle thickness in the branches leading to *Lycodonomorphus bicolor* and *Hydrophis* sp. associated with a terrestrial to aquatic habitat transition, 2) no evolutionary changes in spectacle diameter when species changed between aquatic and terrestrial habitats, and 3) increases in spectacle diameter in several of the branches where snakes became terrestrial from a fossorial ancestor (Fig. 2).

Spectacle thickness and horizontal spectacle diameter showed a significantly positive correlation (*p* = 0.006, *r* = 0.38, phylogenetically independent contrasts), showing that evolutionary increases in eye size was associated with a thicker spectacle.

## Discussion

This study provides a large and phylogenetically very broad examination of the thickness of the snake spectacle in various species with different habitats and activity patterns. Spectacle thickness was found to vary significantly between species. The average thickness ranged between 74 and 244 μm. The thinnest spectacle belonged to a viper (Viperidae), the thickest to a pipe snake (Cylindrophiidae). Species variation of the spectacle has also recently been reported by Van Doorn and Sivak [35] that found that vipers generally have thinner spectacle scales than colubrids. However, that study examined only the keratin layers of the spectacle which are periodically shed along with the skin of the body, whereas this present study was able to include the spectacle itself, the structure which remains permanently fixed to the snake.

The examined snakes were all alcohol-preserved museum specimens. Museum specimens have been validated in other studies [5], and they provided an opportunity to examine a wide range of different species. Some of the specimens had been preserved in ethanol for more than 100 years, however, the quality of the scanning images was remarkable. A previous study displayed no significant difference in spectacle thickness between live and formalin fixed specimens [7]. The specimens examined in this study were fixed in ethanol and studies have shown that morphology of ethanol fixed tissues are comparable to formalin fixed tissues so tissue shrinkage was not considered a concern [36, 37]. In either case all specimens included were handled similarly, so that fixation would not have introduced bias to the study. Some of the examined species had extremely small eyes making it difficult to obtain a representable image as the equipment is based on examinations of the human eye. However, images of even the smallest eye were able to be enlarged on the computer screen using the built-in software so measurements of spectacle thickness were possible. Measurements were made centrally on the spectacle proper. A previous study showed that spectacle thickness is uniform throughout the spectacle proper, but increases in the peripheral region known as the transition zone [7].

The multivariate analysis showed that the variation of spectacle thickness was predicted by taxonomic family and habitat in contrast to spectacle diameter, which was correlated to all three parameters: taxonomic family, habitat and activity pattern. This indicates for example, that diurnal snakes have large eyes but variable spectacle thickness.

When looking at spectacle thickness from a habitat point-of-view, it was found that arboreal and terrestrial snakes had thin spectacles and fossorial and aquatic snakes thick spectacles. This supports the idea that the spectacle has a protective function [3, 35] and that snakes surrounded by water, or living underground need a stronger protective layer than arboreal and terrestrial snakes. From a vision point of view, it could mean that arboreal and terrestrial snakes have a different need for vision than the aquatic and fossorial snakes. Fossorial or burrowing snakes live mainly in the dark and their need for vision is very limited. The aquatic snakes have lost the refractive power of the anterior surface of the eye as a result of a high refractive index of water [38]. On the other hand, terrestrial and arboreal snakes may have developed a thinner spectacle to improve visual acuity, regardless of whether they stem from fossorial [39] or aquatic [40, 41] ancestors. Besides varying needs for vision, another possibility could be the development of a different mechanism of accommodation. Snakes typically focus by moving their rigid lens towards or away from the retina [42] in comparison to mammals where the lens is deformable [43]. It has been stated that the lens of the aquatic dice snake (*Natrix tesselata*) is very flexible compared to other snakes which would be perfectly suited for large degrees of accommodation [44, 38]. The exact mechanisms have, however, not been studied.

Diurnal snakes had significantly larger eyes than nocturnal snakes, a feature contrary to the eyes of mammals, where nocturnal individuals possess the larger eyes. These findings are in accordance with a recent study on colubrid snakes [5]. Snakes with large horizontal spectacle diameter have a larger radius of curvature than snakes with small eyes [5]; and assuming all other factors are equal, the refractive ability ($F=\frac{n^1-n}{r}$, where r = radius of curvature, n = refractive index of medium which light is passing from, n^1^ = refractive index of medium into which light is passing) of a large spectacle is lower than that of a small spectacle [45]. Thus, in diurnal snakes, the lens needs to assume a more powerful refractive role than in nocturnal snakes in order to achieve the same overall refractive power of the eye, as mentioned above in the aquatic dice snake. Furthermore, the cornea could also play a role that is yet to be discovered. Additional investigations of snake vision are required to further elaborate on the refractive power of the snake eye.

The spectacle is found in all snakes, which suggests that it is an adaptive trait, which has been conserved through snake diversification, but its adaptive significance is still unknown. This study shows a large interspecific variation in spectacle morphology among snakes, which may indicate that the spectacle does not serve the same function in all species. We show that ancestral snakes had thick spectacles and were fossorial supporting the idea that the incipient function of the spectacle was eye protection. Since spectacle thickness was secondarily reduced multiple times independently may suggest that an evolutionary trade-off exists between eye protection and other functions, such as vision, resulting in the observed variation in spectacle morphology between extant snake species.

## Conclusion

In conclusion, spectacle thickness appears to correlate with both taxonomy and habitat. The vipers had the significantly thinnest spectacles, both in absolute terms and relative to eye size. The absolute and relatively thickest spectacles belonged to the burrowing snakes of Cylindrophiidae and Uropeltidae, respectively. Aquatic and fossorial snakes had thicker spectacles than terrestrial and arboreal snakes. This knowledge provides additional insight into the evolution of the spectacle of the snake as well as provides further evidence that OCT may be used to examine the anterior eye segment snakes, which may be useful in clinical investigation of cases of ocular disease.
